# Adenosine-mediated immune responses in inflammatory bowel disease

**DOI:** 10.3389/fcell.2024.1429736

**Published:** 2024-08-12

**Authors:** Marta Vuerich, Du Hanh Nguyen, Davide Ferrari, Maria Serena Longhi

**Affiliations:** ^1^ Biomedical Research, Novartis Pharma AG, Basel, Switzerland; ^2^ Department of Anesthesia, Critical Care & Pain Medicine, Beth Israel Deaconess Medical Center and Harvard Medical School, Boston, MA, United States; ^3^ Department of Immunology, University Medical Center Hamburg-Eppendorf, Hamburg, Germany; ^4^ Department of Life Science and Biotechnology, University of Ferrara, Ferrara, Italy

**Keywords:** adenosine, inflammatory bowel disease, inflammation, ATP, adenosine receptors

## Abstract

Extracellular ATP and its derivates mediate a signaling pathway that might be pharmacologically targeted to treat inflammatory conditions. Extracellular adenosine, the product of ATP hydrolysis by ectonucleotidase enzymes, plays a key role in halting inflammation while promoting immune tolerance. The rate-limiting ectoenzyme ENTPD1/CD39 and the ecto-5′-nucleotidase/CD73 are the prototype members of the ectonucleotidase family, being responsible for ATP degradation into immunosuppressive adenosine. The biological effects of adenosine are mediated via adenosine receptors, a family of G protein-coupled receptors largely expressed on immune cells where they modulate innate and adaptive immune responses. Inflammatory bowel disease (IBD) is a serious inflammatory condition of the gastrointestinal tract, associated with substantial morbidity and often refractory to currently available medications. IBD is linked to altered interactions between the gut microbiota and the immune system in genetically predisposed individuals. A wealth of studies conducted in patients and animal models highlighted the role of various adenosine receptors in the modulation of chronic inflammatory diseases like IBD. In this review, we will discuss the most recent findings on adenosine-mediated immune responses in different cell types, with a focus on IBD and its most common manifestations, Crohn’s disease and ulcerative colitis.

## Introduction

Purinergic and adenosine signaling modulate innate and adaptive immune responses virtually in all tissues. Extracellular ATP is released as result of damage or pathological conditions, and rapidly accumulates in inflamed tissues where it is hydrolyzed into adenosine by different enzymes including ectonucleotidases, ectonucleotide pyrophosphatase/phosphodiesterase, alkaline phosphatase and adenylate kinase (Yegutkin and Boison, 2022). Prototype members of the ectonucleotidase family are ENTPD1/CD39, responsible for hydrolyzing ATP and ADP into AMP, and the ecto-5′-nucleotidase/CD73, which converts AMP into adenosine (Allard et al., 2017; Savio et al., 2020). Both ectonucleotidases along with adenosine limit inflammation and restore physiological homeostasis after inflammation-related injury. Additional cell membrane-expressed ectonucleotidases include NTPDase 3 and 8 that preferentially hydrolyze ATP and NTPDase 2 that hydrolyzes ATP only (Kukulski et al., 2005). Cell membrane-expressed ENTPDase 1, 2, 3 and 8 are also present in circulating microparticles in human plasma (Jiang et al., 2014). In the context of inflammatory bowel disease (IBD), a recent study showed that NTPDase8, the dominant enzyme responsible for nucleotide hydrolysis in the gut lumen, protects the intestine from inflammation (Salem et al., 2022). Extracellular nucleosides modulate cellular responses by entering target cells via nucleoside transport mechanisms, based on active transmembrane sodium gradient or facilitated-diffusion carriers (Thorn and Jarvis, 1996). The biological effects of adenosine are mediated also through adenosine receptors (AR), a family of G protein-coupled heptahelical transmembrane receptors, classified into four types: A_1_R, A_2A_R, A_2B_R, and A_3_R (Hasko et al., 2008). Adenosine receptors are largely expressed on immune cells and have been involved in the modulation of inflammatory conditions, including IBD (Thiel et al., 2003; Sitkovsky et al., 2008; Cronstein and Sitkovsky, 2017).

Ulcerative Colitis (UC) and Crohn’s disease (CD) are the most common manifestations of IBD, the incidence of which has increased significantly over the last decades (Ng et al., 2017). IBD has a relapsing/remitting course and often becomes refractory to current therapies. The condition likely results from altered interactions between the gut microbiota and the immune system and is characterized by an imbalance between effector and regulatory immune responses. Mounting evidence has pointed to a pivotal role of adenosine signaling in the modulation of innate and adaptive immunity, proposing it as a therapeutic target in chronic inflammation like in IBD (Vuerich et al., 2020).

## Innate immune responses

### Macrophages

Adenosine immunoregulatory effects on macrophages are supported by clinical and experimental evidence. In patients with ankylosing spondylitis, human M2-like macrophages display dysfunctional A_2A_R that fails to control MMP8 expression, this being associated with disease activity (Sadatpour et al., 2022). In a murine macrophage cell line, exposure to extracellular adenosine, decreases LPS-induced expression of the NADase/ADP-ribosyl-cyclase CD38, as well as the immunomodulatory molecule CD83, while boosting M2 markers and Th2 suppressive cytokines (Devi et al., 2023). In mouse peritoneal macrophages, adenosine suppresses LPS-induced IL-1β release, ROS and nitrite production (Ryzhov et al., 2008), while stimulation of A_2B_R boosts IL-10 production (Nemeth et al., 2005) and contributes to suppression of TNF-α release (Kreckler et al., 2006). In another study, A_2B_R simulation in LPS-activated murine macrophages favors upregulation of the pro-inflammatory cytokine IL-6 (Ryzhov et al., 2008), suggesting that the effects of A_2B_R activation might depend on the experimental setting. In murine peritoneal macrophages, activated A_2A_R suppresses LPS-induced TNF-α release (Kreckler et al., 2006) and promotes its own internalization and degradation by directly boosting lysosomal protease Cathepsin D activity. This latter mechanism is believed to be a strategy for modulating adenosine signaling in inflammatory processes (Skopal et al., 2022).

### Dendritic cells

Adenosine regulates dendritic cells (DCs) upon activation of A_2A_R, A_2B_R and A_1_R. Human DCs tune their response to adenosine also by expressing adenosine deaminase (ADA) that scavenges the nucleoside (Desrosiers et al., 2007).

Exposure to adenosine during LPS-induced maturation, drives human monocyte-derived DC (mDC) differentiation towards an anti-inflammatory phenotype, thus impairing mDC ability to effectively prime CD8^+^ T-cells (Challier et al., 2013). Further, adenosine mediates the recruitment of immature human plasmacytoid dendritic cells (PDCs) to inflammatory sites, via A_1_R signaling. At the infection site, PDCs undergo maturation by decreasing A_1_R expression in favor of A_2A_R, which subsequently limits pro-inflammatory cytokine-release (Schnurr et al., 2004). In human immature DCs (iDCs) and mDCs, chronic A_2A_R stimulation enhances macropinocytotic activity and membrane expression of major histocompatibility complex class-I (MHC-I) and class-II (MHC-II) molecules. Additional evidence has shown that human DCs developed in the presence of adenosine display reduced ability to support T-helper 1 (Th1) polarization. In mDCs, adenosine stimulation inhibits TNF-α and IL-12 release while boosting IL-10 secretion. iDC allostimulatory capacity is reduced upon adenosine exposure (Panther et al., 2003), these data further supporting the immunoregulatory properties of this metabolite.

Akin to human DCs, murine DCs, differentiated in the presence of adenosine, display impaired allostimulatory capacity, and express increased levels of angiogenic and tolerogenic factors, associated with enhanced ability to promote tumors *in vivo* (Novitskiy et al., 2008). A_2A_R signaling limits maturation and inflammatory responses of re-oxygenated murine DCs after hypoxic exposure, critically limiting ischemia reperfusion injury (IRI) (Liu et al., 2015). A_1_R activation suppresses vesicular MHC-I cross-presentation in murine resting DCs (Chen et al., 2008).

The development of Th17-cells from CD4 naïve lymphocytes, has been linked to exposure to DCs-derived and A_2B_R-induced IL-6 (Wilson et al., 2011). In this regard, A_2B_R stimulation, drives differentiation of murine bone marrow-derived DCs into a CD11c^(+)^Gr-1^(+)^subset that promotes Th17 responses (Wilson et al., 2011). Genetic A_2B_R ablation or pharmacological blockade, significantly ameliorates murine autoimmune encephalomyelitis symptoms, by limiting adenosine-mediated IL-6 production by DCs along with Th17-cell differentiation (Wei et al., 2013).

### Neutrophils

Neutrophil recruitment is driven by extracellular ATP and adenosine (Rubenich et al., 2021). Endothelium-derived adenosine prevents activated human neutrophils from damaging the microvasculature at inflammatory loci, by controlling their response to complement-coated beads (Kubersky et al., 1989). Adenosine produced by CD73 expressing human neutrophils, limits excessive endothelial paracellular permeability, this avoiding potentially deleterious disturbance in vascular function during inflammation (Lennon et al., 1998). Neutrophil-derived CD73 provides adenosine also in the intestinal lumen, triggering IL-6 release by epithelial cells, which leads to neutrophil activation and degranulation, an event that supports anti-microbial immune responses (Sitaraman et al., 2001).

Adenosine exposure can have antithetical effects on neutrophil function, depending on the adenosine receptor subtype involved. Rapid and beneficial effects of adenosine administration have been reported in COVID-19-related acute respiratory syndrome and pneumonia (Correale et al., 2020; Spiess et al., 2021), which are characterized by heightened neutrophil responses (Li et al., 2023). Upon stimulation of A_1_R, low adenosine concentrations promote neutrophil adherence to endothelium, chemotaxis towards the inflammation site and FcγR function. Higher adenosine levels, which may occur at sites of tissue damage, inhibit adherence, FcγR function, degranulation, and generation of toxic oxygen metabolites (Salmon and Cronstein, 1990; Cronstein et al., 1992; Visser et al., 2000).

In human and murine neutrophils, A_2A_R activation suppresses pro-inflammatory cytokine production (McColl et al., 2006) and activates the cyclooxygenase-2 pathway, leading to anti-inflammatory prostaglandin E2 release. This phenomenon could be part of an early regulatory mechanism that favors anti-inflammatory activities at inflamed sites (Cadieux et al., 2005).

In human neutrophils, A_2A_R expression and ligand affinity are tightly influenced by the surrounding environment. *In vitro* exposure of human neutrophils to LPS or Th1 cytokines boosts A_2A_R expression (Fortin et al., 2006). On the other hand, A_2A_R-ligand affinity is reduced in neutrophils isolated from sepsis patients, this explaining the limited regulatory effects of adenosine in this setting (Kreth et al., 2009). Moreover, A_2A_R stimulation in neutrophils isolated from murine models or patients with sepsis, fails to suppress cell death, slows aging, and promotes a N2 phenotype, when compared to cells obtained from controls (Lovaszi et al., 2022). In human polymorphonuclear leukocytes, exposure to pro-inflammatory conditions limits A_2A_R expression by boosting miRNA-214, miRNA-15 and miRNA-16.These miRNAs could serve as useful markers to identify patients more susceptible to severe inflammation (Heyn et al., 2012).

In a murine model of IRI, A_2A_R activation in neutrophils and CD4^+^ T-cells, improves animal conditions by limiting inflammation (Sharma et al., 2010). In LPS-induced acute lung injury, downregulation of neutrophil trafficking and responses is noted after A_1_R activation and is associated with beneficial effects (Ngamsri et al., 2010). Notably, the oxygen-induced iatrogenic exacerbation of acute lung injury is linked to the suppression of A_2A_R-mediated lung tissue-protecting pathway. Intratracheal injection of a selective A_2A_R agonist effectively restores the protective effects of endogenous adenosine on lungs (Thiel et al., 2005).

An immunoregulatory role has been noted for the low affinity A_2B_R that suppresses oxidase activity in murine neutrophils (van der Hoeven et al., 2011) *in vitro* and in an *in vivo* model of acute kidney injury, by curbing neutrophil-dependent TNF-α release (Grenz et al., 2012). Similar results have been obtained in murine models of peritonitis and peritonitis-related sepsis, where selective inhibition of CXCR4 and CXCR7 ameliorates disease, limiting neutrophil infiltration at the inflammatory site in an A_2B_R-mediated manner (Ngamsri et al., 2020).

Human and murine neutrophils can actively contribute to the metabolic control of the inflammatory milieu, by releasing ATP through connexin 43 hemichannels in a protein/phosphatase-A-dependent manner (Eltzschig et al., 2006).

A_3_R and P2Y2R play an important role in neutrophil chemotaxis. Experiments conducted in murine models of sepsis, revealed that both receptors are closely involved in neutrophil migration to the lungs, suggesting that pharmaceutical approaches targeting these receptors might help in controlling acute lung tissue injury in this condition (Inoue et al., 2008). Human neutrophil recruitment is also positively modulated by A_3_R (Butler et al., 2012).

### NK/NKT-cells

In inflammatory conditions, a counteracting mechanism to prevent deleterious inflammatory responses, is represented by upregulation of adenosine signaling mediators in NK-cells.

In sickle cell disease (SDC), the widely disseminated microvascular IRI boosts A_2A_R expression in human and murine CD4+invariant-natural-killer-T (iNKT)-cells, via nuclear factor-kappaB (NF-κB)-signaling (Lin et al., 2013). A SCD phase-1 trial of the A_2A_R agonist regadenoson, resulted in decreased activation of iNKT-cells without significant side effects (Field et al., 2013). *Ex vivo* activated iNKT-cells from healthy donors, upregulate anti-inflammatory purinergic mediators like A_2A_R, CD39 and CD73 (Yu et al., 2018). iNKT-cells from SCD patients also express elevated levels of CD39 (Yu et al., 2018). In another study, CD39^+^ NK-cells were found to be induced by IL-15 and to display more effective cytotoxicity when compared to CD39^−^ NK-cells. There is evidence that A_2A_R stimulation opposes IL-15-induced generation of human CD39^+^ NK-cells, by blocking IL-15 signaling (Kang et al., 2023). In the same cells, A_2A_R is also associated with IL-4 production, which is inhibited by receptor blockade. These data are further corroborated by the evidence that A_2A_R-deficient mice are characterized by a marked decrease in IL-4, IL-10 and TGF-β, while concomitantly displaying increase in IFN-γ (Nowak et al., 2010). Importantly, adenosine and its analogues have been found to limit NK cell lytic activity (Raskovalova et al., 2005). In murine models of liver IRI, the CD1d-dependent NKT-cell inflammatory response, is abrogated by A_2A_R activation (Lappas et al., 2006). Similarly, the NKT-cell-mediated liver injury associated to Concanavalin-A-induced acute hepatitis, is abolished by the A_2A_R agonist CGS21680 and exacerbated in A_2A_R-deficient mice (Subramanian et al., 2014). Adoptive transfer of murine iNKT and NK-cells with induced A_2A_R activation into SCD mice improves pulmonary function and prevents exacerbation of hypoxia-reoxygenation-induced pulmonary injury (Wallace and Linden, 2010).

### Mast cells

Mast cells play an important role in asthma, where their activity is strongly boosted by adenosine. In ADA-deficient mice that naturally develop lung inflammation reminiscent of asthma, ADA enzyme therapy has beneficial effects, preventing accumulation of adenosine and mast cell degranulation (Zhong et al., 2001). In mouse, airway response to aerosolized adenosine largely depends on mast cells and A_3_R activation (Tilley et al., 2003).

A_3_R can activate human mast cells independently of IgE and triggers upregulation of growth factors, cytokines and chemokines, by coupling to the cellular target of receptor mimetic basic secretagogues Gi3 (Baram et al., 2010). The pro-inflammatory effects of adenosine, in murine mast cells, are phosphoinositide 3-kinase gamma (PI3Kgamma)-dependent (Laffargue et al., 2002). Data generated in human A_3_R-expressing mice, revealed that the human A_3_R response *per se* is not sufficient to activate PI3Kgamma-dependent signaling pathways, and to mediate adenosine effects (Yamano et al., 2005). The impact of adenosine on human mast cells also depends on A_2B_R that triggers release of Th2 cytokines, including IL-4 and IL-13, which, in turn, induce IgE synthesis by B lymphocytes (Ryzhov et al., 2004).

### Th1/Tc1-cells

The effect of adenosine on murine Th1 and Tc1 responses has been largely investigated. Adenosine receptor activation inhibits type I cytokine secretion, especially IL-2, limiting expansion *in vivo* (Erdmann et al., 2005). In mouse, A_2A_R stimulation, can inhibit nonalcoholic steatohepatitis development by reducing Th17-cell expansion and IL-17-induced JNK-dependent lipotoxicity (Alchera et al., 2017). Inosine administration can also reduce inflammation and mortality in a model of endotoxemia in mice, through abrogation of pro-inflammatory Th1 cytokine production by macrophages and splenocytes. The effect is partially reversed by blockade of A_1_R and A_2A_R (Hasko et al., 2000). Further supporting these studies is the evidence that *Leishmania infantum* parasites stimulate A_2A_R signaling, limiting the development of Th1 adaptive immunity and favoring parasitic colonization (Lima et al., 2017).

### Th17-cells

Adenosine signaling can trigger different responses in human and murine Th17-cells, these depending on the adenosine receptor involved.

In PBMCs from asthma patients, *A*_*2A*_ *R* mRNA levels correlate positively with Treg- and negatively with Th17-cell markers and asthma severity. Accordingly, in a murine model of OVA-induced lung inflammation, pharmacological A_2A_R stimulation triggers Treg responses, while inhibiting Th17 lung infiltration (Wang et al., 2018). Low levels of CD39 and A_2A_R expression have been detected in Th17-cells and found to support inflammation in juvenile patients with autoimmune liver disease (Liberal et al., 2016).

A_2B_R is upregulated in DCs from MS patients, and its occupancy by adenosine triggers IL-6 secretion that promotes Th17-cell differentiation. In line with this, pharmacological A_2B_R blockade alleviates murine experimental autoimmune encephalomyelitis, via inhibition of the DCs/IL-6-Th17 axis (Wilson et al., 2011; Wei et al., 2013).

In *Trichinella spiralis* infection, A_2A_R upregulation accelerates post-infectious irritable bowel syndrome by promoting polarization of CD4^+^ T-cells into Th17 lymphocytes (Dong et al., 2022).

### Th2-cells

In human and mouse, Th2 responses are driven upon activation of different adenosine receptors. In IL-33-activated murine bone-marrow-derived group 2 innate lymphoid cells, A_2B_R stimulation suppresses IL-13 and IL-5 levels while A_2A_R activation triggers IL-5 production without affecting IL-13 release (Csoka et al., 2018). In a cockroach allergen model of murine asthma-like pulmonary inflammation, systemic or myeloid A_2B_R deletion, has beneficial effects associated with decreased Th2-type airways responses, i.e., reduction of lung IL-4, IL-5, and IL-13 (Belikoff et al., 2012).

A_1_R removal in ADA-deficient mice, leads to enhanced pulmonary inflammation-related damage, associated with increased expression of the Th2 cytokines IL-4 and IL-13 in the lung (Sun et al., 2005).

### Tregs

Human and murine Tregs express CD39 that initiates ATP/ADP hydrolysis to ultimately generate adenosine. Expression of A_2A_R by Tregs enables adenosine to act in an autocrine manner, favoring the maintenance of the Treg pool. A_2A_R stimulation further boosts CD39 and CD73 expression in Tregs isolated from septic mice (Bao et al., 2016). Notably, A_2A_R stimulation effectively counteracts Treg deficiency that drives autoimmunity in scurfy mice (He et al., 2017).

**FIGURE 1:**
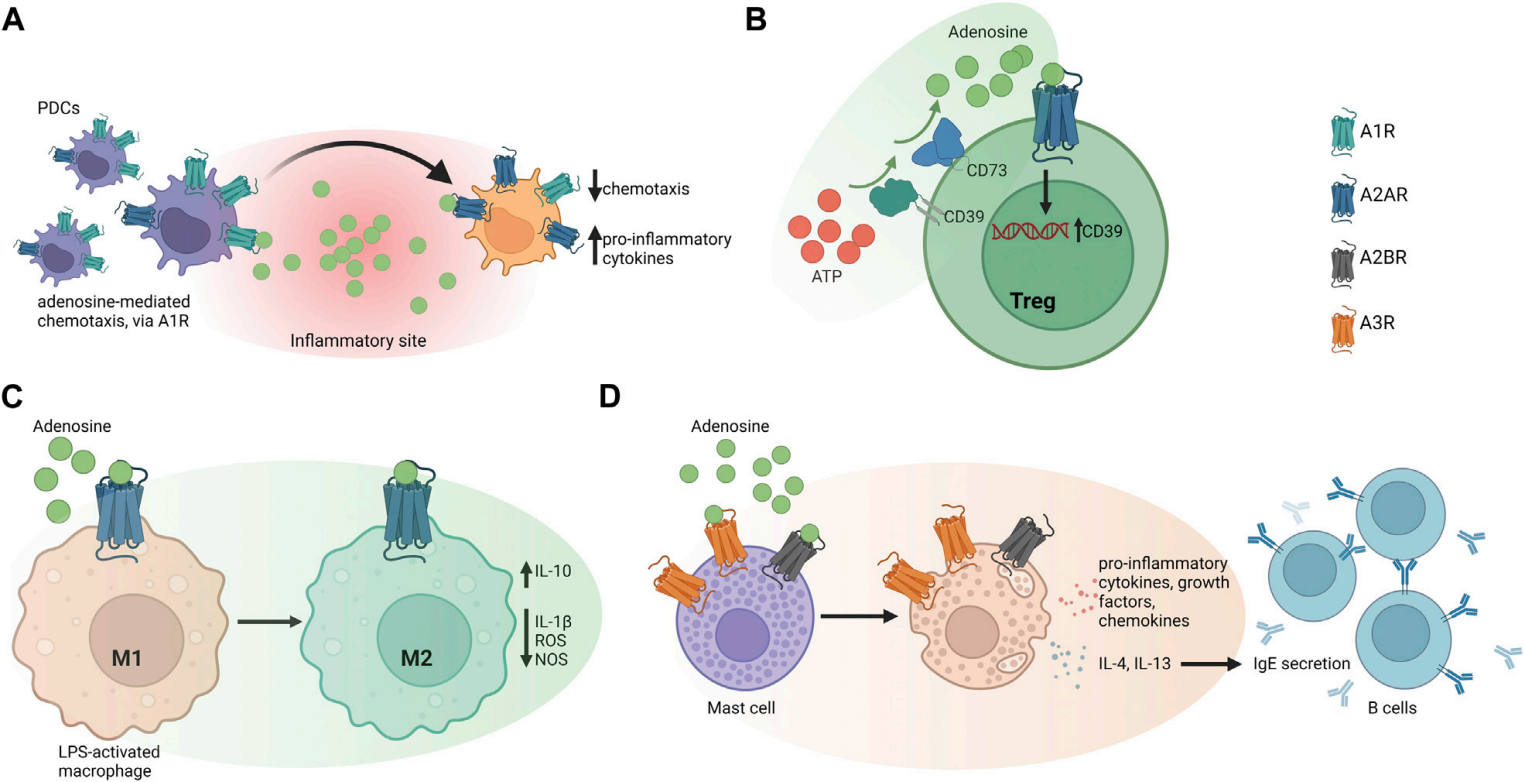
Adenosine receptor signaling in different immune cell types. **(A)** Adenosine mediates immature human plasmacytoid dendritic cells (PDCs) recruitment to inflammatory sites, via A_1_ receptor (A_1_R) signaling. At the infection site, PDCs undergo maturation by decreasing A_1_R expression, in favor of A_2A_R, which limits pro-inflammatory cytokine release. **(B)** Human and murine Tregs express CD39 that initiates ATP/ADP hydrolysis to ultimately produce adenosine. Expression of A_2A_R by Tregs enables adenosine to act in an autocrine manner that might favor the maintenance of the Treg-cell pool. **(C)** In mouse peritoneal macrophages, adenosine suppresses LPS-induced IL-1β release, ROS, and nitrite production. A_2B_R stimulation boosts IL-10 production and suppresses pro-inflammatory responses **(D)** A_3_R can activate human mast cells independently of IgE and triggers upregulation of growth factors, cytokines, and chemokines. The effect of adenosine on human mast cells also depends on A_2B_R, which promotes release of Th2 cytokines like IL-4 and IL-13 to induce IgE synthesis in B lymphocytes.

Adoptively transferred CD73-deficient or A_2A_R-deficient Tregs, fail to confer protection in a murine IRI model, while pharmacological A_2A_R stimulation augments the protective effect of wild-type and CD73-deficient Tregs (Kinsey et al., 2012). In OVA-induced lung inflammation, pharmacological A_2A_R activation promotes anti-inflammatory Treg responses (Wang et al., 2018). The same receptor mediates Treg homing to the site of inflammation in a murine model of experimental autoimmune uveoretinitis, and in cells from human uveitis patients, *in vitro* (Peters et al., 2023). In contrast, A_2B_R inhibits Treg cell infiltration in a murine heterotopic tracheal model of bronchiolitis obliterans (Zhao et al., 2010), postulating a different role of this receptor in this disease setting. Figure 1 represents adenosine receptor signaling in immune cells.

Supplementary Table S1 summarizes the effects of adenosine receptor activation in different cell types and disease models.

### Adenosine signaling in IBD

Several studies support the beneficial effects of adenosine in IBD. Increasing evidence suggests that agonism or inhibition of adenosine receptors A_2A_R, A_2B_R and A_3_R might be exploited therapeutically (Vuerich et al., 2020).

### A_2A_R

A_2A_R signaling is often impaired in IBD. In the colonic mucosa of UC patients, A_2A_R is post-transcriptionally downregulated by miR-16, this limiting anti-inflammatory inhibition of NF-κB signaling pathway (Tian et al., 2016).

The immunomodulatory role of A_2A_R in inflammation depends on its effect on T-cells, in which expression levels are subset-specific. A_2A_R expression is higher in CD4^+^ than CD8^+^ T-cells; however, upon T-cell activation, the receptor levels are mainly boosted in the latter (Koshiba et al., 1999). During CD8^+^ and CD4^+^ T-cell activation, A_2A_R signaling inhibits cytotoxicity and cytokine-release activity, while only marginally affecting proliferation (Ohta et al., 2009). A_2A_R stimulation favors also CD4^+^CD25^high^FoxP3^+^ Treg expansion, and this is associated with increased CTLA-4 levels and heightened suppression (Ohta et al., 2012). Blood and lamina propria of CD patients display reduced frequencies of suppressive CD39^+^ Th17-cells, an immunoregulatory Th17-cell population resistant to the effects of adenosine, as a result of heightened ADA and decreased A_2A_R levels (Longhi et al., 2014).

A_2A_R-deficient mice are more susceptible to tissue damage induced by sub-threshold doses of inflammatory stimuli (Ohta and Sitkovsky, 2001). These findings are corroborated by the evidence that administration of the A_2A_R selective agonist ATL-146e reduces intestinal mucosa inflammation by limiting leukocyte infiltration and pro-inflammatory cytokine release in murine experimental models of IBD (Odashima et al., 2005). Studies have shown that electroacupuncture inhibits visceral pain in IBD mice by boosting A_2A_R, A_1_R and A_3_R levels, while inhibiting A_2B_R in colon tissue (Hou et al., 2019). In murine models of chronic colitis, administration of ADA inhibitors results in beneficial effects through enhanced activation of A_2A_R and A_3_R (Antonioli et al., 2010). The immunosuppressive properties of A_2A_R are noted also in murine cancer models (Sitkovsky et al., 2008). Genetic deletion of A_2A_R in host animals favors rejection of immunogenic tumors and anti-cancer immunity is restored only upon administration of A_2A_R antagonists, or by silencing the receptor via siRNA pretreatment on T-cells (Ohta et al., 2006).

Despite robust evidence of an immunosuppressive role in cancer and experimental colitis models, a study showed that increased A_2A_R expression as a consequence of *T. spiralis* infection, promotes post-infectious irritable bowel syndrome by inducing Th17-cell polarization in mouse (Dong et al., 2022); this suggesting a different role of this receptor in specific disease settings.

A_2A_R inhibitory effects on colonic motility are enhanced in the presence of bowel inflammation, as noted when colonic longitudinal muscle preparations from healthy rats or with experimental colitis are used (Antonioli et al., 2006). *Ex vivo* treatment with a highly polar, perorally nonabsorbable A_2A_R selective agonist, significantly ameliorates acetylcholine-induced contractions in ileum/jejunum preparations from inflamed rats (El-Tayeb et al., 2011). In comparable settings, A_2A_R stimulation, or A_2B_R blockade, prevents inflammation-induced contractile disturbance (Michael et al., 2010).

In various experimental models of colitis in rats, administration of the A_2A_R agonist polydeoxyribonucleotide restores tissue structural integrity by reducing inflammatory cytokine expression, myeloperoxidase activity, and malondialdehyde. Accordingly, all the beneficial effects are abrogated by concomitant administration of A_2A_R antagonist DMPX (Pallio et al., 2016).

### A_2B_R

The pro-inflammatory, low affinity A_2B_R is the predominant adenosine receptor expressed in the colon.

There is evidence that A_2B_R depletion ameliorates the course of experimental colitis in different murine models (Kolachala V. L. et al., 2008). In DSS-induced colitis, administration of the A_2B_R antagonist ATL-801 prevents weight loss and suppresses the inflammatory infiltrate in the colonic mucosa (Kolachala V. et al., 2008). In a mouse model of IRI and in a cell model of acute hypoxia, A_2B_R antagonism improved the intestinal epithelial structure and barrier function, further supporting the pro-inflammatory role of A_2B_R activation (Yang et al., 2014).

Conversely, other studies provided evidence of a selective role for epithelial A_2B_R signaling in attenuating colonic inflammation in the context of DSS-induced colitis (Aherne et al., 2015). Similarly, additional investigations in the setting of DSS colitis in mice with partial genetic deficiency of netrin-1, a molecule implicated in the modulation of leukocyte trafficking, indicate a role for A_2B_R in mediating netrin-1 protective effects (Aherne et al., 2012). A_2B_R-mediated immunosuppression is critically involved in cancer immune escaping. A_2B_R pharmacological inactivation or genetic depletion prevents effector T-cell inhibition by the hypoxic tumor microenvironment, thus facilitating tumor rejection (Lukashev et al., 2007).

### A_3_R

The Gi protein-associated A_3_R has been proposed having both anti- and pro-inflammatory properties. A study by Rybaczyk and coll showed a positive correlation between A_3_R downregulation and acute inflammatory score, disease chronicity and purine genes dysregulation, when considering colonic mucosal biopsies or PBMCs obtained from CD, UC or control subjects (Rybaczyk et al., 2009). In line with this, in colonic epithelial cells from UC patients, A_3_R activation mitigates pro-inflammatory cytokine production, by curbing NF-κB signaling (Ren et al., 2014; Ren et al., 2020). In another study on colonic epithelial cells from UC patients, A_3_R is downregulated and its expression levels inversely correlate with those of mir-206, a post-translational regulator associated with disease histological activity (Minacapelli et al., 2019) and linked to exacerbation of DSS colitis in mice (Wu et al., 2017). In mice with DSS-induced colitis downregulation of A_3_R is partially limited following miR-206-antagomir treatment (Wu et al., 2017).

**FIGURE 2:**
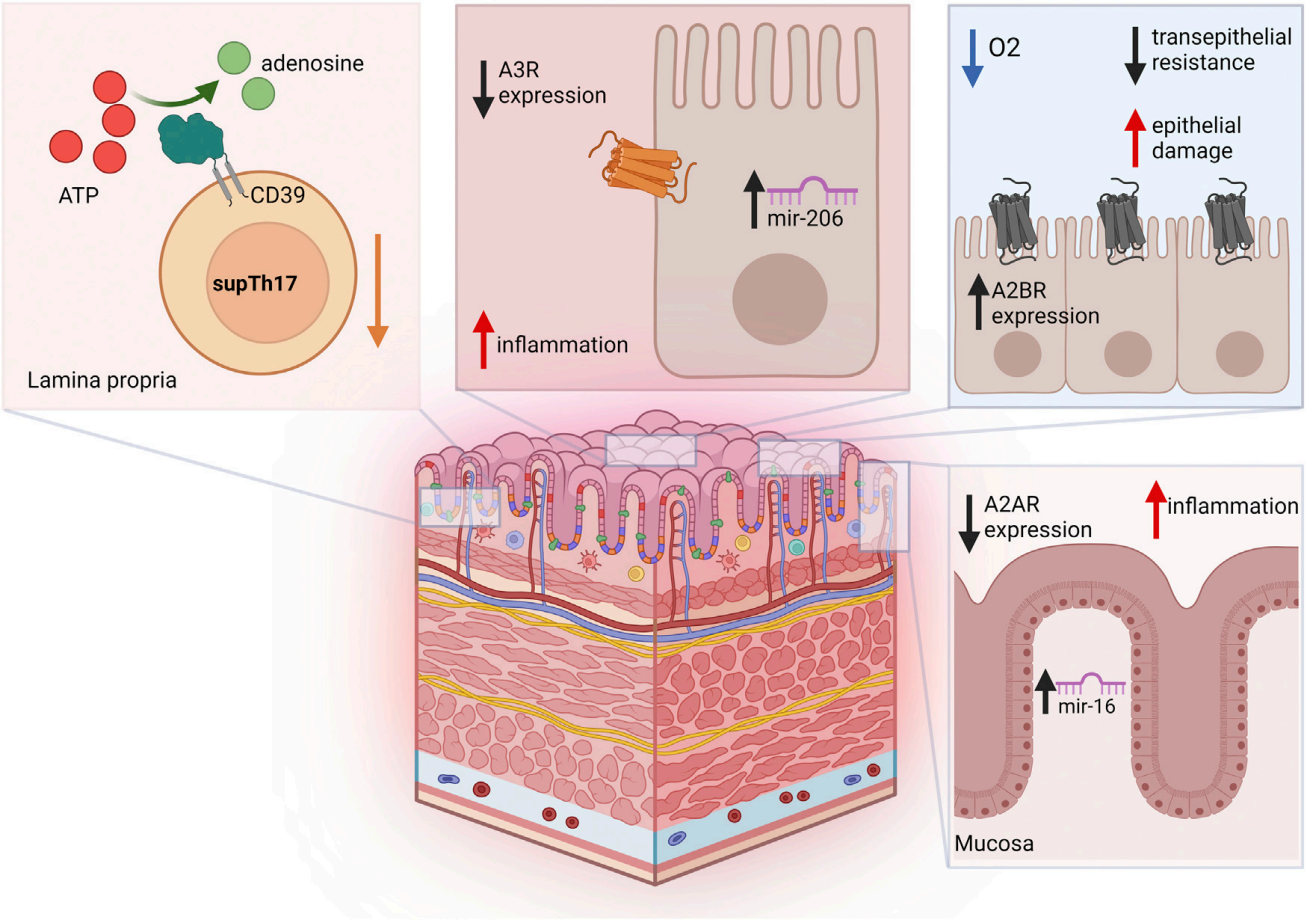
Adenosine-related pathways in IBD. Blood and lamina propria of Crohn’s disease patients are characterized by reduced frequencies of suppressive CD39^+^ Th17-cells (supTh17), an immunoregulatory Th17-cell population that is impaired in IBD. In colon epithelial cells from UC patients, A_3_R is downregulated, and its expression levels inversely correlate with mir-206. Increase in A_2B_R levels during intestinal IRI and acute hypoxia has been implicated in reduced transepithelial resistance and increased epithelial damage. In the colonic mucosa of UC patients, A_2A_R is post-transcriptionally downregulated by miR-16, this limiting A_2A_R anti-inflammatory effects.

Treatment of chronic colitis induced by 2,4,6-trinitrobenzene sulfonic acid using the A_3_R agonist N (6)-(3-iodobenzyl)-adenosine-5-N-methyluronamide (IB-MECA), further supports the protective role of this adenosine receptor. The treatment contains the colitis-induced upregulation of several pro-inflammatory genes including *P2X1R*, *P2X4R*, *P2X7R*, *P2Y2R*, and *P2Y6R*, downregulated *P2X2R*, *P2Y1R*, and *P2Y4R*, significantly reducing the inflammatory score and ameliorating disease course in animals (Guzman et al., 2006). Additional investigations showed that the A_3_R agonist AR170 contrasts inflammatory cell infiltration of the colon and decreases pro-inflammatory cytokine levels also in 2,4-dinitrobenzene sulfonic acid-induced colitis in rats (Antonioli et al., 2020). In contrast to the above studies, genetic deletion of A_3_R resulted in disrupted intestinal transit, conferring protection against DSS colitis (Ren et al., 2011). In line with these findings, Ochaion and coll reported upregulation of A_3_R levels in PBMCs from patients affected by rheumatoid arthritis, psoriasis, and CD. A_3_R upregulation was found to be related to NF-κB and CREB protein pathways (Ochaion et al., 2009). Figure 2 shows adenosine-related pathways in IBD.

## Future perspectives and conclusions

We have briefly discussed how adenosine signaling modulates immune function and dictates outcomes in experimental and human IBD. How to fine-tuning adenosine-mediated immune responses to selectively halt inflammation at the sites of interest, while inducing homeostasis remains unclear. The development of purinergic signaling-based therapies, in combination with conventional treatments, could help promoting and maintaining immunotolerance in IBD and other immune-mediated chronic conditions.
