# Supplementary material for: Adenosine-mediated immune responses in inflammatory bowel disease
Source: Front Cell Dev Biol. 2024 Aug 12;12:1429736. doi: 10.3389/fcell.2024.1429736 (PMC11345147; doi:10.3389/fcell.2024.1429736)
Supplement: Supplementary file 1 [file Table1.pdf]

Supplementary Table 1. Adenosine receptor expression and activation effects.

Adenosine receptor	Immune cell population	Species/Experimental settings	Effect of receptor activation (otherwise differently specified)	References
**A_1R**	Dendritic cells	Human plasmacytoid dendritic cells (PDCs)	Cell recruitment to infection site	Schnurr et al., 2004
		Murine resting DCs	Suppression of vesicular MHC-I cross-presentation	Chen et al., 2008
	Neutrophils	Human neutrophils	Favors cell adherence to endothelium, chemotaxis towards the inflammation site and FcγR function	Salmon et al., 1990
		Human polymorphonuclear leukocytes	Exposure to pro-inflammatory conditions limits $A_{2A}R$ expression by boosting miRNA-214, miRNA-15 and miRNA-16	Heyn et al., 2012
		LPS-induced acute lung injury in mouse	Downregulation of neutrophil trafficking and responses, leading to a beneficial effect	Ngamsri et al., 2010
	Th2-cells	A_1R removal in ADA deficient mice	Enhanced pulmonary inflammation due to increased expression of the Th2 cytokines IL-4 and IL-13 in the lung	Sun et al., 2005
**$A_{2A}R$**	Macrophages	Murine macrophage cell line and peritoneal macrophages	Decrease in LPS-induced pro-inflammatory response, upregulation of M2 markers	Sadatpour et al., 2022; Devi et al., 2023
	Neutrophils	Sepsis patients or murine sepsis models	$A_{2A}R$ expression and ligand affinity are reduced, failing to mediate adenosine immunosuppressive activity. $A_{2A}R$ stimulation fails to suppress cell death, slows aging, and promotes a N2 phenotype	Kreth et al., 2009; Lovaszi et al., 2022
		Murine model of ischemia reperfusion injury (IRI)	Limitation of inflammation and improvement of animal condition	Sharma et al., 2010
	Dendritic cells	Human PDCs	Limitation of pro-inflammatory cytokines release at infection site	Schnurr et al., 2004
		Human immature DCs (iDCs) and monocyte-derived DCs (mDCs)	Enhanced macropinocytotic activity and increased membrane expression of major histocompatibility complex class-I (MHC-I) and class-II (MHC-II) molecules	Panther et al., 2003
		Murine DCs in IRI model	Limited maturation and inflammatory responses of re-oxygenated murine DCs after hypoxic exposure	Liu et al., 2015
	NK/NK T-cells	Human and murine invariant NK-cells (iNKT); human and murine models of sickle cell disease (SDC). NK-cells in murine models of IRI and Concanavalin-A induced hepatitis	Beneficial effect leading to decreased iNKT-cell activation	Field et al., 2013; Lappas et al., 2006; Wallace et al., 2010
		Ex vivo activated human iNKT-cells	$A_{2A}R$ upregulated upon cell activation	Yu et al., 2018
		Human and murine NK-cells	Anti-inflammatory cytokines production in mouse; inhibition of NK-cell differentiation through blockade of IL-15 signaling in humans	Kang et al., 2023; Nowak et al., 2010
	Th1/Tc1-cells	Murine model of nonalcoholic steatohepatitis	Beneficial effect, reduced Th17-cell expansion and JNK-dependent lipotoxicity	Alchera et al., 2017
		Murine models of *Leishmania infantum*	Limited development of Th1 adaptive immunity	Lima et al., 2017
	Th17-cells	Asthma patients, murine models of OVA-induced lung inflammation, autoimmune liver disease patients	Immunosuppressive role, disrupted in disease	Wang et al., 2018; Liberal et al., 2016
	Treg-cells	Human and murine Treg-cells	Autocrine promotion of adenosine-mediated regulatory phenotype	Bao et al., 2016
		Scurfy mouse model	Effective counteraction of Treg deficiency that drives autoimmunity	He et al., 2017
		Murine IRI model	$A_{2A}R$ deficient Tregs fail to confer protection during inflammation	Kinsey et al., 2012
		Ova-induced lung inflammation	Enhanced Treg responses	Lima et al., 2017

		Murine model of experimental autoimmune uveoretinitis; cells from uveitis patients	Treg migration to the site of inflammation *in vivo* or *in vitro*	Zhao et al., 2010
		Murine Tregs	$CD4^{+}CD25^{high}FoxP3^{+}$ Treg expansion	Ohta et al., 2012
		Human $CD4^{+}$ and $CD8^{+}$ T-cells	During T-cell activation, $A_{2A}R$ signaling inhibits cytotoxicity and cytokine-release activity	Ohta et al., 2009
	Group 2 innate lymphoid cells	Murine, *in vitro*	Suppression of IL-13 and IL-15 release	Csoka et al., 2018
**$A_{2B}R$**	Myeloid cells	Mouse peritoneal macrophages	IL-10 and IL-6 production, suppression of TNF-α release	Rhyzov et al., 2008 ; Nemeth et al., 2005 ; Kreckler et al., 2006
		Cockroach allergen model of murine asthma-like pulmonary inflammation	Systemic receptor deletion in myeloid cells is beneficial due to decrease in Th2 airway responses	Belikoff et al., 2012
	Dendritic cells	Murine bone marrow-derived DCs, murine autoimmune encephalomyelitis model	Differentiation into a CD11c(+) Gr-1(+), IL-6 releasing subset, that promotes Th17-cell differentiation and immune responses	Wilson et al., 2011; Wei et al., 2013
	Neutrophils	*In vitro* and *in vivo* model of acute kidney injury and murine models of peritonitis and peritonitis-related sepsis	Inhibition of TNF-α release	Grenz et al., 2012; Ngamsri et al., 2020
	Mast cells	Human mast cells	Release of Th2 cytokines	Rhyzov et al., 2004
	Th17-cells	*Trichinella spiralis* infection - murine model	Receptor upregulation accelerates post-infection IBD by promoting Th17-cell differentiation	Dong et al., 2022
	Treg-cells	Murine heterotopic tracheal model of bronchiolitis obliterans	Inhibition of Treg-cell infiltration at the inflammatory site	Zhao et al., 2010
	Group 2 innate lymphoid cells	Murine, *in vitro*	IL-15 releases	Csoka et al., 2018
**A_3R**	Neutrophils	Human and murine neutrophils	Cell migration to the lungs	Inoue et al., 2008; Butler et al., 2012
	Mast cells	Murine models of airway inflammation	Modulation of adenosine responses	Tilley et al., 2003
